# Telocytes: new insight into the pathogenesis of gallstone disease

**DOI:** 10.1111/jcmm.12057

**Published:** 2013-04-04

**Authors:** Andrzej Matyja, Krzysztof Gil, Artur Pasternak, Krystyna Sztefko, Mariusz Gajda, Krzysztof A Tomaszewski, Maciej Matyja, Jerzy A Walocha, Jan Kulig, Piotr Thor

**Affiliations:** a First Department of General, Oncological and Gastrointestinal Surgery, Jagiellonian University Medical CollegeKrakow, Poland; b Department of Pathophysiology, Jagiellonian University Medical CollegeKrakow, Poland; c Department of Anatomy, Jagiellonian University Medical CollegeKrakow, Poland; d Department of Clinical Biochemistry, Jagiellonian University Medical CollegeKrakow, Poland; e Department of Histology, Jagiellonian University Medical CollegeKrakow, Poland

**Keywords:** telocytes, interstitial Cajal-like cells, gallstones, cholesterol saturation index, bile lithogenicity

## Abstract

The major mechanisms of gallstone formation include biliary cholesterol hypersecretion, supersaturation and crystallization, mucus hypersecretion, gel formation and bile stasis. Gallbladder hypomotility seems to be a key event that triggers the precipitation of cholesterol microcrystals from supersaturated lithogenic bile. Telocytes, a new type of interstitial cells, have been recently identified in many organs, including gallbladder. Considering telocyte functions, it is presumed that these cells might be involved in the signalling processes. The purpose of this study was to correlate the quantity of telocytes in the gallbladder with the lithogenicity of bile. Gallbladder specimens were collected from 24 patients who underwent elective laparoscopic cholecystectomy for symptomatic gallstone disease. The control group consisted of 25 consecutive patients who received elective treatment for pancreatic head tumours. Telocytes were visualized in paraffin sections of gallbladders with double immunofluorescence using primary antibodies against c-Kit (anti-CD117) and anti-mast cell tryptase. Cholesterol, phospholipid and bile acid levels were measured in gallbladder bile. The number of telocytes in the gallbladder wall was significantly lower in the study group than that in the control group (3.03 ± 1.43 *versus* 6.34 ± 1.66 cell/field of view in the muscularis propria, *P* < 0.001) and correlated with a significant increase in the cholesterol saturation index. The glycocholic and taurocholic acid levels were significantly elevated in the control subjects compared with the study group. The results suggest that bile composition may play an important role in the reduction in telocytes density in the gallbladder.

## Introduction

Gallstone disease occurs in 10–15% in adults in Europe and the United States of America and is one of the most common and most expensive of the digestive disorders that require hospital admission 1, 2. In developed countries, cholesterol gallstones account for 80–90% of the gallstones found at cholecystectomy 3. The aetiology of gallstone disease is considered to be multifactorial, with roles for both genetic and environmental factors 4, 5. The main mechanisms of cholesterol lithogenesis include biliary cholesterol hypersecretion, supersaturation and crystallization, stone formation and growth, bile stasis and mucus hypersecretion and gel formation within the gallbladder. Gallbladder hypomotility may be a key factor in the pathogenesis of cholelithiasis because it allows time for cholesterol microcrystals to precipitate from lithogenic bile that is supersaturated with cholesterol 6–8. The impaired gallbladder motility observed *in vivo* in a subset of patients with gallstones correlates well with the decreased *in vivo* contractility of both gallbladder strips and isolated gallbladder smooth muscle cells 9, 10.

In recent years, our understanding of the physiology and regulatory mechanisms of smooth muscle tissue and the role of interstitium has been enhanced by the study of a population of newly described cells, the so-called interstitial Cajal – like cells (ICLCs). Such name was adopted, as these cells were thought to represent the phenotypic similarity (at least by means of immunohistochemistry, as they expressed c-Kit marker – CD117) to the archetypal, enteric interstitial cell of Cajal (ICC). Characteristic feature of ICLCs was the small, triangular-shaped body, and the presence of several very long prolongations. Multiple research teams have identified such cells in various tissues outside of the gut 11, including the pancreas 12, lungs 13, heart 14, skin 15, skeletal muscle 16, parotid gland 17, meninges and the choroid plexus 18, ureter, urethra, urinary bladder 19, 20, kidney 21, blood vessels 22, male 23, 24 and female reproductive organs 25–28, mammary glands 29, 30 and placenta 31. During the last 5 years Prof. Popescu with his teammates examined such cells and little by little, it became clear that the ultrastructure of ICLCs was different from that of ICCs, and that the difference between these cells was not only semantic, as they have different ultrastructural and immunophenotypic patterns, and therefore these cells should be functionally distinct as well 32.

To distinguish ICLCs and to avoid confusion from other interstitial cell type, *i.e*. fibroblasts, fibrocytes, fibroblast-like cells, or mesenchymal cells, a new name was coined as telocytes (TCs) to replace ICLCs, according to the morphological characteristics through immunohistochemistry and electron microscopy 32. Because in Greek affix ‘telos’ means ‘goal,’ ‘end’, and ‘fulfillment’, the extremely long but thin prolongations of TCs was named as telopodes (Tps). The concept of TCs has been currently adopted by other laboratories.

Telocytes were recently discovered in the wall of the human gallbladder by Hinescu *et al*. 33 and in bile ducts by Ahmadi *et al*. 34. To date, there has been no direct evidence that TCs are directly involved in the regulation of gallbladder motility. However, a decrease in the density of TCs in the muscle layer of the gallbladder could be hypothetically related to bile stasis and thus might contribute to gallstone formation, because TCs are suggested to be involved in signalling processes 14, 16. Previously, we reported a significant decrease in c-Kit – positive cells density (TCs) in the gallbladder wall in patients suffering from gallstone disease 35. The purpose of this study was to determine whether telocyte loss was related to the degree of bile lithogenicity, which was expressed as a lithogenic index (cholesterol saturation index, CSI), or to the difference in bile salt profiles in patients who suffer from cholecystolithiasis and those who are not affected by this disease.

## Materials and methods

### Subjects

Twenty-four consecutive patients with symptomatic gallstone disease were scheduled for elective surgery (laparoscopic cholecystectomy) and selected for the study group (eight men, mean age 52.4 ± 11.7 years; 16 women, mean age 54.9 ± 16.1 years). Patients with gallstones had detectable calculi in the gallbladder on ultrasound examination before the operation. They presented with mild, recurrent episodes of biliary colic. None of these patients had associated choledocholithiasis or acute cholecystitis. The control group consisted of 25 consecutive patients (11 men, mean age 62.0 ± 7.6 years; 14 women, mean age 61.0 ± 9.8 years), who were electively treated for pancreatic head tumours and had no pre- or intraoperative signs of cholelithiasis or jaundice. Pancreaticoduodenectomy was performed according to the standard Whipple procedure or the pylorus-sparing Traverso-Longmire technique in patients with resectable lesions. For patients with non-resectable lesions, bypass (gastroenterostomy) was carried out for palliative purposes. Gallbladders that were not affected by primary tumours and did not contain any gallstones were removed. Serum bilirubin levels were measured pre-operatively and were normal in both groups. All patients were surgically treated in the First Department of General, Oncological and Gastrointestinal Surgery at the Jagiellonian University Medical College from 2010 to 2011.

### Ethical approval

The study was conducted in accordance with the moral, ethical, regulatory and scientific principles governing clinical research. All surgical samples were retrieved with the approval of the Jagiellonian University Bioethical Committee using procedures that conformed to the Declaration of Helsinki guidelines (protocol number – KBET/30/B/2010).

### Tissue processing

Tissue samples from fresh cholecystectomy specimens were collected and rinsed thoroughly with PBS (phosphate-buffered saline, 0.01 M, pH = 7.4), fixed in 4% phosphate-buffered paraformaldehyde, routinely processed and embedded in paraffin. Serial sections were cut and mounted on poly-l-lysine-coated glass slides and stained with haematoxylin and eosin (H&E) for routine histopathology.

### Immunohistochemistry

Indirect double immunofluorescence after heat-induced epitope retrieval was used to allow the simultaneous visualization of two antigens. The sections were incubated overnight with a mixture of rabbit polyclonal anti-c-Kit antibody (anti-CD117; A4502; Dako, Glostrup, Denmark; diluted 1:150) and mouse monoclonal anti-mast cell tryptase antibody (M7052; Dako; 1:800) or a mouse monoclonal anti-CD34 antibody (NCL-ENDO; Novocastra, Newcastle, UK; 1:50). The sections were rinsed in PBS and incubated for 1 hr at room temperature with a mixture of a Cy3-conjugated goat anti-rabbit antibody (111-165-144; Jackson ImmunoResearch, West Grove, PA, USA; 165-144; 1:600) and a biotinylated goat anti-mouse antibody (115-065-146; Jackson IR; 1:600). After washing in PBS, the slides were incubated with DTAF-conjugated streptavidin (016-010-084; Jackson IR; 1:500 in PBS) for 1 hr. After a final rinse in PBS, the nuclei were counterstained with DAPI (D9542; Sigma-Aldrich, St. Louis, MO, USA; 1:30,000) for 30 sec. The sections were mounted in Vectashield medium (H-1000; Vector Laboratories, Burlingame, CA, USA) to minimize fluorophores photobleaching.

### Microscopic examination and quantification of telocytes

Slides were examined using an Olympus BX50 epifluorescence microscope (Olympus, Tokyo, Japan) equipped with an Olympus DP71 digital CCD camera. The use of mast cell tryptase staining enabled c-Kit-positive mast cells to be distinguished from telocytes. The distribution of TCs in the gallbladder corpus was quantitatively assessed. TCs were considered to be c-Kit positive and tryptase negative concurrently. These cells were counted in 10 consecutive high-power fields (400×). The data are expressed as the mean number of cells per 1 field of view (FOV) of gallbladder muscularis propria. The thickness of the muscularis propria in the same region of the gallbladder was also measured using image analysis software (Multiscan v.18, Computers Scanning System, Warsaw, Poland).

### Gallbladder bile collection, sample analysis and CSI calculation

Bile specimens were obtained from all subjects by needle aspiration of the gallbladder during surgery. Aliquots of bile samples were stored at −70°C prior to biliary lipid composition analysis. The cholesterol and phospholipid concentrations and bile acid levels in the bile samples were determined.

The bile samples were extracted on SEP-PACK-NH_2_ columns (500 mg; Waters, Milford, MA, USA). Each column was first activated using 6 ml of n-hexane; then, 0.1 ml of centrifuged bile sample was applied to the column, and the flow-through was discarded. The samples were eluted in three 1-ml volumes of a chloroform-isopropanol mixture (3:1, v/v), followed by three 1-ml volumes of methanol. The eluted fractions from each column were collected and dried at 50°C under nitrogen.

The dry residues were reconstituted in 0.5 ml of isopropanol and mixed vigorously. Cholesterol (Randox Laboratories Ltd., Crumlin, UK) and phospholipid (Wako Chemicals, Neuss, Germany) concentrations were measured by enzymatic methods. The intra- and interassay coefficient of variations were 3% and 4.8% for cholesterol and 5% and 6% for phospholipids, respectively. All determinations were performed with a Cobas-Bio analyser (Roche Analytical Instruments, Inc., Nutley, NJ, USA).

Individual bile acids were measured by reverse-phase high-performance liquid chromatography with an isocratic solvent system (Waters). Prior to chromatographic separation, bile acids were extracted from bile samples using SEP-PACK C18 columns (Waters). All columns were activated using 5 ml of methanol and 5 ml of water, after which 0.1 ml of bile mixed with phosphate buffer (0.07 mmol/l, pH 7.0) and 0.1 ml of internal standard were applied. Then, the columns were washed with 10 ml of water, 3 ml of 10% acetonitrile and another 10 ml of water. The bile acids were eluted in 3 ml of methanol. The eluates were dried at 37°C under nitrogen, and dry residues were re-dissolved in 1 ml of an acetonitrile-water mixture (1:1, v/v). Each sample was filtered using a Millex GN filter (13 mm) and separated chromatographically using an XTERRA RPC-18 column (18.5 × 3.9 × 150 mm, Waters) with detection at 200 nm.

The mobile phase (flow rate 2 ml/min.) contained 10% acetonitrile in a mixture of methanol and 0.1 M monobasic potassium phosphate (60:40, v/v, pH 4.50). Before use, the solvent was filtered through a 0.45 μm filter (type HV, Millipore, Bedford, MA, USA). An elution profile of conjugated bile acid standards (Sigma-Aldrich) was obtained by injecting 20 μl of a standard bile acid mixture in methanol that contained glycocholic acid (1.640 mmol/l), taurocholic acid (1.488 mmol/l), glycochenodeoxycholic acid (1.696 mmol/l), glycodeoxycholic acid (1.696 mmol/l), taurochenodeoxycholic acid (1.532 mmol/l) and taurodeoxycholic acid (1.532 mmol/l).

Based on cholesterol, phospholipid and total bile acid concentrations, the CSI was obtained from Carey's critical tables 36, 37. Bile samples with a CSI equal to 1 or more were considered supersaturated.

### Data analysis and statistical evaluation

The data are expressed as the mean and standard deviation (SD). The results were analysed using a one-way analysis of variance (anova), followed by a *post-hoc* LSD test. Pearson's correlation test was used to examine the relationship between continuous variables. *P* values less than 0.05 were considered to indicate statistical significance. All statistical analyses were performed using STATISTICA 9.0 software (StatSoft, Tulsa, OK, USA).

## Results

### Histopathological findings

**Fig. 1:**
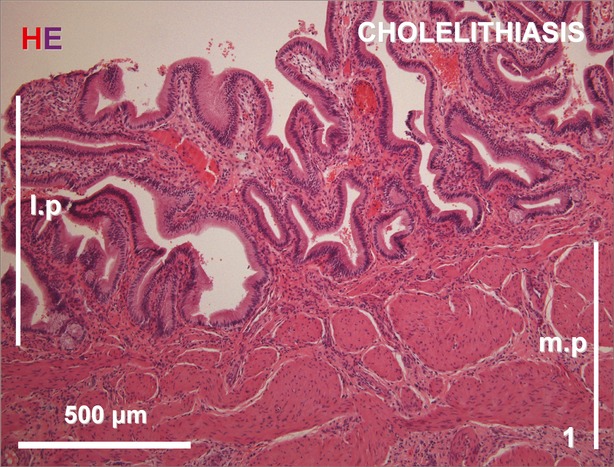
Cross section of the gallbladder wall from the cholelithiatic group showing mild infiltration with inflammatory cells and a thickened muscular layer. H&E staining. l.p., lamina propria; m.p., muscularis propria.

The histopathological evaluations showed chronic cholecystitis of varying intensity in both groups of patients. The inflammation was assessed and designated as mild, moderate or severe. In the study group, severe inflammation was predominant (Fig. 1). In contrast, only mild to intermediate inflammation was observed in the control group. The thickness of the gallbladder muscularis propria was significantly increased in the study group compared with the control group (4453 ± 597 *versus* 3610 ± 346 μm; *P* < 0.01).

**Fig. 2:**
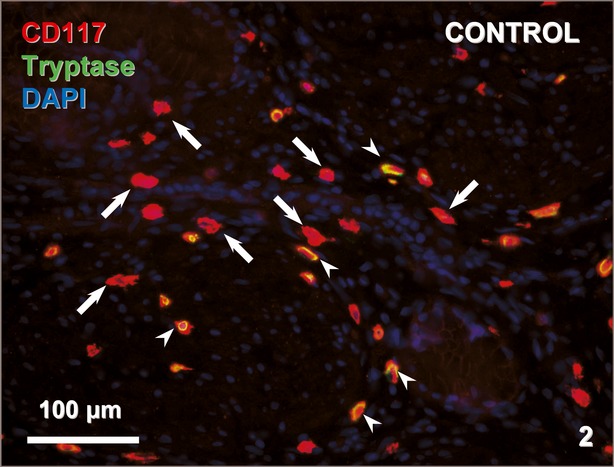
Cross section of the gallbladder wall of a control patient stained for CD117 (red) and tryptase (green). The nuclei are counterstained with DAPI (blue). CD117-positive/tryptase-negative TCs (arrows) and CD117-positive/tryptase-positive mast cells (arrowheads) are indicated.

**Fig. 3:**
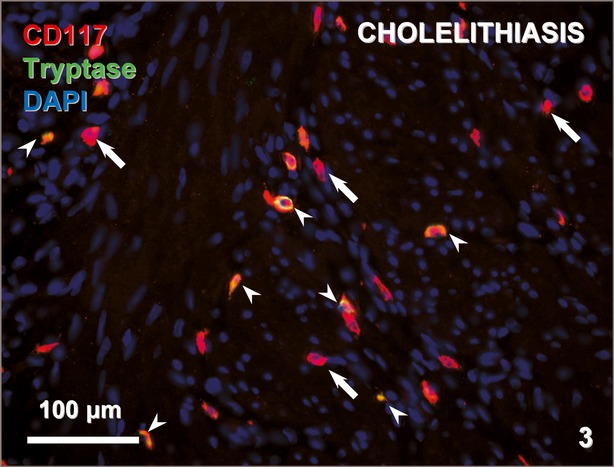
Cross sections of the gallbladder wall of a cholelithiatic patient stained for CD117 (red) and tryptase (green). The nuclei are counterstained with DAPI (blue). CD117-positive/tryptase-negative TCs (arrows) and CD117-positive/tryptase-positive mast cells (arrowheads) are indicated.

The number of mast cells in the gallbladder wall was evaluated in specimens immunostained for tryptase. Mast cells were present in all layers of the gallbladder wall and predominantly localized to the lamina propria (Figs 2 and 3). The total number of mast cells in the gallbladder wall was significantly higher in patients with gallstones than in control subjects (224 ± 41 *versus* 161 ± 37, respectively; *P* < 0.05). In immunostained slides, c-Kit and tryptase double-positive mast cells were generally round or oval shaped, with a centrally located nucleus.

The c-Kit-positive/mast cell tryptase-negative cells were considered to be telocytes. We found them predominantly located in the corpus, but these cells were also observed in the gallbladder fundus and neck. TCs had a centrally located nucleus and were mostly fusiform in shape with small branches, that were visible in some sections; however, sparse, round tryptase/c-Kit-positive cells were also present. Numerous TCs were detected, mostly in the muscularis propria, and some TCs were observed in the connective tissue separating the smooth muscle bundles (Fig. 1).

The number of TCs in the gallbladder wall corpus was significantly lower in the study group than that in the control group (3.03 ± 1.43 *versus* 6.34 ± 1.66 cell/FOV in the muscularis propria; *P* < 0.001) (Figs 2 and 3).

CD-34-positive cells were visualized in the gallbladder; however, they appeared to be mainly of vascular origin. CD-34-positive cells were rarely encountered in the muscularis propria. These CD-34-positive cells were c-Kit negative concurrently.

### Bile composition analysis

**Fig. 4:**
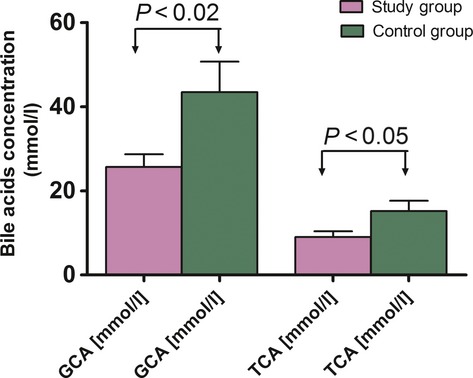
Concentrations of glycocholic acid (GCA) and taurocholic acid (TCA) in gallbladder bile from patients with cholecystolithiasis (*n* = 24) and controls (*n* = 25). The data are expressed as the mean values ± SD.

The study revealed a significant decrease in the mean concentrations of glycocholic and taurocholic acids in the bile from patients with cholelithiasis compared with that in the controls (*P < 0.02* and *P < 0.05*, respectively) (Fig. 4). There was also a positive correlation between the TC count and the concentrations of glycocholic (*r* = 0.45, *P* = 0.039) and taurocholic (*r* = 0.32, *P* = 0.05) acids.

**Fig. 5:**
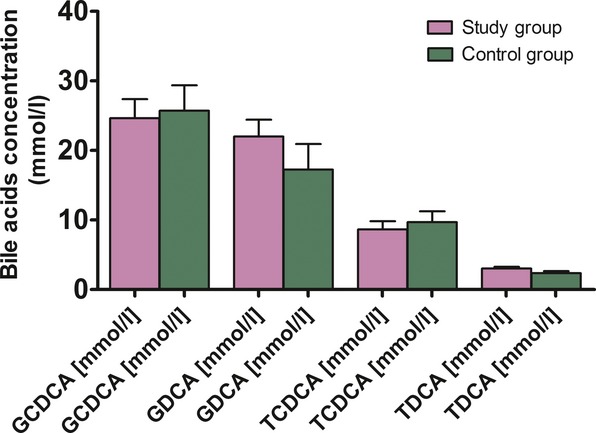
Bile acid concentrations of glycochenodeoxycholic acid (GCDCA), glycodeoxycholic acid (GDCA), taurochenodeoxycholic acid (TCDCA) and taurodeoxycholic acid (TDCA) in gallbladder bile from patients with cholecystolithiasis (*n* = 24) and controls (*n* = 25). The data are expressed as the mean values ± SD.

**Fig. 6:**
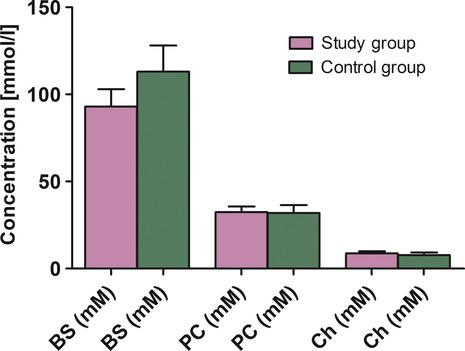
Concentration of total bile acids (BS), phospholipids (PC) and cholesterol (Ch) in gallbladder bile from patients with cholecystolithiasis (*n* = 24) and controls (*n* = 25). The data are expressed as the mean values ± SD.

No significant differences in the concentrations of the other bile acids examined (glycochenodeoxycholic, glycodeoxycholic, taurochenodeoxycholic and taurodeoxycholic acid) were observed between the two groups of patients (Fig. 5). There were also no significant differences in the concentrations of cholesterol, bile salts or phospholipids in the bile between the two groups (Fig. 6).

**Fig. 7:**
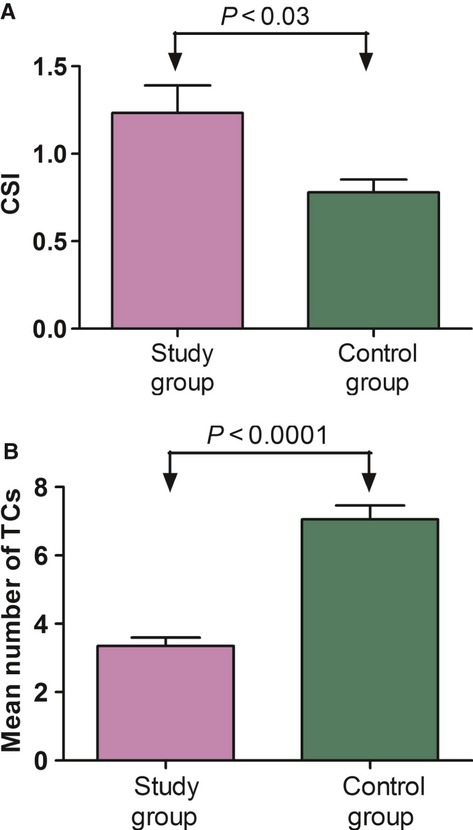
Cholesterol saturation index (CSI) (**A** – upper part) and the number of TCs (**B** – lower part) in patients with cholecystolithiasis (*n* = 24) and controls (*n* = 25). The data are expressed as the mean values ± SD.

The calculated CSI was significantly higher in patients with cholecystolithiasis (1.23 ± 0.84) than that in the controls (0.78 ± 0.33) (*P < 0.05*). Moreover, the results revealed an important negative correlation (*r* = −0.62, *P* = 0.001) between the CSI and the TC count in the gallbladder wall (Fig. 7A and B).

## Discussion

Multiple factors are responsible for gallstone formation. Regardless of cholesterol supersaturation, hydrophobic bile salts, pronucleating proteins and mucus hypersecretion with gel formation in the gallbladder 38–41, gallbladder dysmotility may be a ‘triggering’ event in the pathogenesis of cholesterol gallstones, providing the time necessary for the precipitation of cholesterol microcrystals from bile supersaturated with cholesterol and their subsequent growth to macroscopic stones 39, 42. Gallbladder motility involves multiple regulatory mechanisms, including smooth muscle and enteric nervous circuit activity as well as, possibly, the recently described gallbladder interstitial cells—telocytes.

Telocyte is a unique type of interstitial cell with specific prolongations named Tps 32, 43. TCs have been described by electron microscopy in several cavitary and non-cavitary organs of humans and mammalians forming a 3D network anchored by hetero- and homocellular junctions. Tps are an alternation of thin segments (podomers) and dilated segments (podoms). Podomers are very thin (less than 0.2 μm), often below the resolving power of light microscopy, explaining the fact that TCs have been overlooked up to now. The interstitium (stroma) is in most of the cases seen as a connecting ‘device’ for the specific structures of an organ. Interstitial cells are usually perceived as being mainly (or even, only) fibroblasts. However, fibroblasts synthesize connective tissue matrix, *i.e*. collagen. The distinction between TCs and fibroblasts is obvious because they have different ultrastructure and phenotype. Therefore, their functions should be mostly different: TCs—intercellular signalling (connections), but fibroblasts—collagen synthesis. In other words, TCs are ‘more’ functionally oriented, and fibroblasts are ‘more’ structurally oriented, responsible for fibrosis. There are some clear ultrastructural features that differentiate TCs from fibroblasts. For instance, the general aspect of TC is of a small oval (piriform/spindle/triangular/stellate)-shaped cellular body, containing a nucleus surrounded by a small amount of cytoplasm. The shape of the cell body depends on the number of Tps. Fibroblast cell body is pleomorphic. TC cellular body average dimensions are, as measured on EM images, 9.3 ± 3.2 μm (min. 6.3 μm; max. 16.4 μm). Fibroblast nucleus is typically euchromatic, but TC nucleus is mostly heterochromatic. Mitochondria represent only 2% of cell body volume and the Golgi complex is small in TC. Fibroblasts Golgi complex is prominent and the rough endoplasmic reticulum is very well developed (usually 5–12%) of cell volume 44, 45.

Telocytes in the human gallbladder were first described in gastrointestinal stromal tumours that originated from cells in the gallbladder wall with an ICC-like phenotype that expressed CD117 46, 47. TCs were subsequently identified in several animals. Lavoie *et al*. 48 reported the existence of TCs in the guinea pig gallbladder and described their potential function in electrical coupling in smooth muscle cells. Balemba *et al*. 49 described the role of mitochondrial Ca^2+^ handling in the activity of smooth muscle cells and TCs and underscored the role of TCs in regulating the tone of the gallbladder in guinea pigs. Huang *et al*. 50 used whole-mount preparations of the gallbladder and extrahepatic guinea pig biliary ducts to identify TCs and suspected the role of such cells in propagation of spontaneous rhythmicity and excitability of the gallbladder. Xu *et al*. 51 demonstrated that the cholecystokinin-A receptor on TCs was essential for gallbladder muscle contractions in guinea pig muscle strips. Sun *et al*. 52 successfully identified TCs in the gallbladder of CD1 mice using confocal microscopy of whole-mount flat preparations.

The published data on TCs in the human gallbladder remain limited. Hinescu *et al*. 33 reported that TCs in the human gallbladder typically appeared individually or in small clusters of two to three cells. According to these authors, the TCs were located in the lamina propria, with some very close to the epithelium, and in the connective tissue spaces between bundles of smooth muscle cells. TCs accounted for 5.5% of the subepithelial cells in the gallbladder wall. Meanwhile, Ahmadi *et al*. 34 identified c-Kit-positive cells with characteristic morphology in the subepithelial and muscular layers of the gallbladder and extrahepatic bile ducts (which are denser than those of the gallbladder) running parallel to circular smooth muscle fibres, forming a cellular network. These authors did not find TCs in intrahepatic bile ducts.

In this study, TCs within gallbladder wall cross sections were identified based on anti-c-Kit and anti-tryptase double immunofluorescence to distinguish such cells from mast cells. TCs were predominantly located in the muscularis propria. We expected that the decrease in TC density in the study group would be related to hypertrophy of the muscularis propria. However, there was a significant (20%) increase in the thickness of the gallbladder wall in the study group compared with the control group, while the TC count was reduced by 50%. Surprisingly, this significant loss of TCs in the gallbladder wall in patients with gallstones was strongly correlated with an increased CSI.

Even under physiological conditions, the cholesterol content in the aqueous solution of gallbladder bile is relatively high because cholesterol is incorporated into mixed micelles, together with bile salts and phospholipids. When the bile cholesterol levels increase or the secretion of solubilizing bile salt is diminished, the solution becomes supersaturated. In this case, excess cholesterol is stored in vesicles (spheres composed of cholesterol and phospholipids, with no bile salts), provided that enough phospholipid is available. When relatively low amounts of phospholipids are present, cholesterol crystal formation occurs, leading to gallstone formation 8, 39, 53. Moreover, cholesterol crystallization is also promoted by hydrophobic bile salts (*e.g*. chenodeoxycholate and deoxycholate) and phospholipids with unsaturated acyl chains 54. Human bile contains a mixture of both hydrophobic (*e.g*. deoxycholate) and hydrophilic (*e.g*. ursodeoxycholic) acids, and thus, the final hydrophobicity of the bile salts depends on the relative contents of these components 55–57. Therefore, the biliary bile salt composition affects human gallstone formation, which opens several therapeutic possibilities 58–61. Our results showed that there were no significant differences between the two groups of patients in the mean concentrations of total bile acids, phospholipids, or cholesterol in vesicular bile, except for the lower mean concentrations of glycocholic and taurocholic acid in the patient group. The lower amounts of these mostly hydrophilic bile acids were associated with the increased lithogenicity index (CSI) in the study group. Interestingly, in patients with gallstones, a significant positive correlation between the mean number of TCs and the concentrations of glycocholic and taurocholic acids was found. We conclude that glycocholic and taurocholic acids are somehow protective to TCs. However, it is not clear whether this is an artefact of the statistical analysis or whether TCs are indeed preserved by these acids, and the exact mechanism of this possible protective effect should be examined further, including in an experimental model.

We acknowledge that possible mechanisms underlying the destructive influence of bile on TCs remain speculative rather than empiric, as reports on the role of TCs in gallstone pathophysiology are sparse. Some insights were provided by Hu *et al*. 62 who demonstrated that the expression of c-Kit mRNA and protein in the gallbladder wall was significantly decreased in the gallbladders of guinea pigs fed a high-cholesterol diet. A study by Xu and Shaffer 9 reported that gallbladder hypomotility was impaired by the increased bile cholesterol level. A study by Lavoie *et al*. 63 proved that excess cholesterol in the smooth muscle of the gallbladder attenuates the ability of the muscle to contract as a result of changes in signal transduction and ion channel activity, decoupled membrane receptor–ligand interactions and disturbances in contractile protein activity. Moreover, in a subsequent study on guinea pigs fed a lithogenic diet, Lavoie *et al*. 48 reported cholesterol accumulation in gallbladder smooth muscles in the plasma membrane, especially membrane caveolae, leading to a decrease in membrane fluidity and a subsequent change in rhythmic electric activity. Hypertrophy was also detected in the muscularis propria, and the contractile response to an agonist was decreased. It is not yet known whether such mechanisms could influence TC activity as well. Furthermore, TCs seem not to be directly involved in the pacemaking mechanism, but rather possibly act as modulators of the contractility *e.g*. through the released exosomes/shedding vesicles 45. Elements of the TC network are interacting with each other (homo-cellular connections) as well as with other cell types (hetero-cellular connections). As reported in the heart, the homocellular junctions occur at both podomeric and podomic level, either side to side (presumably for exchanging information) or end to end (probably for relaying, passing on information). Heterocellular junctions are encountered a between TCs and myocytes, fibroblasts, mast cells, macrophages, pericytes, endothelial or Schwann cells. TCs are integrating such cellular types into a complex 3D network. These networks are providing both structural and functional support for long-distance signalling 14, 16, 64.

Another important mechanism underlying TCs loss concerns the chronic inflammatory processes involving the gallbladder wall. Portincasa *et al*. 8 described impaired gallbladder motility caused by mild inflammation. Indeed, we observed inflammatory infiltration, predominantly localized in the lamina propria, in the gallbladders of patients with gallstones, which was associated with a significant increase in the mast cell count. However, as we reported previously, TC loss does not correlate with inflammatory grade or mast cell count 35.

Nevertheless, other indirect inflammatory effects on gallbladder TCs cannot be excluded. For example, apoptotic mechanisms leading to the loss of gallbladder TCs should be considered. The apoptosis might be caused by multiple mechanisms, including chronic inflammatory reactions 65, and it is possible that the reduced number of TCs in the inflamed gallbladder wall in patients with gallstones could be caused by an imbalance between the apoptosis and regeneration of TCs. Finally, the reduced number of TCs might be primarily caused by the trans-differentiation of TCs precursor cells, as reported by Torihashi *et al*. in the smooth muscles in the gut 66.

This study established that altered bile composition in patients with cholelithiasis may influence TCs within the gallbladder muscle. We conclude that a reduction in TC number may be a consequence of the toxicity of the supersaturated bile, while some other bile components (glycocholic and taurocholic acids) may exert protective effects on TCs and thus possibly influence the mechanisms regulating gallbladder and extrahepatic bile duct motility.
